# Southern Dental Association

**Published:** 1885-08

**Authors:** 


### Reports of Society Meetings.

### SOUTHERN DENTAL ASSOCIATION.

SEVENTEENTH ANNUAL MEETING, AT NEW ORLEANS.

REPORTED FOR THE INDEPENDENT PRACTITIONER, BY MRS. M. W. J.

(Concluded from page 372.)

#### THIRD DAY.

Thursday, April 2d, being Dentists' Day at the World's Exposition, no regular sessions were held, the day being devoted to sight-seeing, speech-making and dining, the members being guests of the dentists of New Orleans. A delightful day was passed, and all returned, refreshed and invigorated, to their labors on Friday.

#### FOURTH DAY.

Friday was set apart for Clinics and the Exhibition of Appliances. Mr. J. W. Selby, of the S. S. White Dental Manufacturing Co., furnished the hall with all the necessary chairs, brackets, spittoons, etc., and several clinics were given, commencing at 9 A. M.

*Dr. Wm. N. Morrison*—Illustrated the method of filling root-canals, as explained in his paper already given. A committee had been appointed to prepare the teeth for filling. They had selected those with the most tortuous roots, abounding in sharp angles and crooked twists and turns, without crown cavities, and had imbedded them in blocks of plaster of paris in such manner as to afford no clew to the form of the roots. The work of Dr. Morrison was closely watched, and many were the predictions of failure. He drilled an opening through the center of each crown, not much larger than a pin-hole, through which he removed the contents of the root-canals, and inserted his fillings of gold wire imbedded in a solution of gutta-percha in chloroform. When the work was finished the plaster was broken away and the teeth critically examined. There was not one failure, and the verdict was unanimous that Dr. Morrison had done all that he claimed to be able to do. Whether anyone else could do it remained an open question.

*Dr. E. Parmly Brown*—Gave a clinic with the electric mallet and spring pluggers of his own device, so fashioned and tempered as to have a very elastic spring above the point, obviating the danger of crumbling frail margins of enamel. The separation for knuckling was obtained by the force of the gold, driving the teeth apart at the point of contact without any preparatory separation. A tooth of which a large portion of the crown had been filed away was rebuilt in gold, restoring the contour of the cusps and masticating surface.

*Dr. Bonwill*—Gave a clinic with his mechanical engine and mallet, of which the reporter failed to obtain the details.

At 3.30 P. M. the meeting was called to order, that those having appliances to exhibit might have an opportunity of explaining their merits.

The chairman of the committee, Dr. H. J. McKellops, stated that there were many interesting and valuable inventions and methods on exhibition; that time and trouble had been expended in getting them together, and he hoped that all would feel interested in them.

Among the articles may be named a simple and ingenious articulator, by Dr. Westmoreland, of Columbus, Miss.; a lathe head-piece (name of inventor not attached); a tooth-powder-bottle stopper, patented by Dr. C. E. Kells, Jr., of New Orleans; a regulating spring, of piano wire, with a triple coil in the middle, increasing the tension, and doing away with jack-screws, or the plate which steadies the wire on the Coffin plan. This is the invention of Dr. E. S. Talbot, of Chicago. A new mouth-piece for the administration of nitrous oxide gas, by Dr. Miles, of Charleston; by Dr. Williams, of Ky., a new motor power for engines and lathes, which gives great power and increased rapidity of revolution; by Dr. J. W. Smith, Newport, R. I., paper disks, with sandpaper rim; by Dr. B. S. Byrnes, Memphis, Tenn., a regulating appliance, consisting only of a very thin elastic band of 22 carat gold, worked around the first molar, or most available posterior teeth at either end, and indented with the interstices between the teeth, the constant, gentle pressure of the elastic band causing the anterior teeth to yield, the space gained being retained by straightening the band, shortening it as much as needed, and applying as before. Casts were shown indicating wonderful results in a very short time. This appliance was highly commended by Dr. McKellops. Dr. J. A. Robinson exhibited rubber plates lined with vulcanizable gold, preventing the contact of the rubber with the mucous membrane, thus avoiding its injurious results. Dr. Edwards exhibited specimens of very light elastic black rubber plates, made by a new method, which produces a highly polished surface, finished when it leaves the flask. Dr. J. R. Walker showed a modification of the Coffin plate, the spring so adapted as to be attached to the front teeth to draw them into place while the arch is being expanded by the action of the spring. Dr. Bonwill exhibited his mechanical mallet and engine. Dr. Morrison showed some interesting specimens of Japanese dental art. Also a gold crown that had been presented by him to the Southern Dental Association fifteen years ago, anterior to the present patents on gold crowns. Mr. J. W. Lambert exhibited samples of Listerine and menthated camphor, explaining their virtues. McKesson & Robbins, through Mr. Wm. Evans, exhibited samples of the muriate and oleate of cocaine, explaining their application in dentistry.

### EVENING SESSION.

Dr. B. H. Catching, 3d Vice-president, in the chair.

The report of the Committee on Dental Literature was called for.

*Dr. B. H. Catching*—Read an interesting paper, emphasizing the importance of the literature of a profession to its growth and influence, the disadvantages under which a man labors who "thinks he knows it all;" showing the favorable status of the literature of dentistry as compared with that of the other professions, but deploring its shortcomings in some branches; namely, the text-books of materia medica and therapeutics, the ignorance of the average practitioner who is satisfied with carbolic acid, creosote, tannin, and arsenic, and the slowness of our periodical literature as compared with the electric facilities for the current news of the day, concluding that the monthly journal was too slow for the age, and that a weekly journal was needed.

*Dr. E. Parmly Brown*—In a few telling words, deplored the fact that so many from the North had failed to visit the World's Exposition, and eulogized the welcome he had received. His remarks were warmly applauded.

*Dr. E. S. Chisholm*—Read a second paper on Dental Literature. He spoke of the Index of the Cosmos, recently published, as affording an index to dental literature generally, in the great variety of topics presented. He said that while in our journals we are distinctive, in our text-books we rely too much upon medicine for both authors and teachers. That though in many of the branches we owe our fundamental knowledge to medicine, we have in our specialty got beyond borrowing so freely, and are returning with usury. Our own text-books are not deficient in quantity, whatever may be said of quality, eighteen new volumes having been added to the list within the year, either entirely new, or in thoroughly revised and re-written new editions. He spoke of the war waged upon advertising pages, but claimed that it was a gain, a free gift to the reader, telling him where to find what he most needed. He spoke of guarding the threshold of our colleges against the admission of illiterate, ignorant young men, who sought only the means of making money without honest labor.

The two papers elicited a lengthy and interesting discussion of the subject.

*Dr. Spalding*—Deplored the inferior quality of much of our professional literature and the incorrect precepts of our text-books, claiming that we depended upon medical men to write and teach dental students that of which they themselves are ignorant, and urging that dentistry bring forth from its own ranks men to teach and to write for its own students.

*Dr. Robinson*—Stated that in the dental college of the Michigan University all the members of the faculty were practical dentists.

*Dr. Catching*—Regretted that no samples of dental literature were ever exhibited at associations; it should be a part of the work of the Committee on Dental Literature to procure copies of all new works from the publishers, to form part of their report at each annual meeting.

*Prof. J. Taft*—Reviewed the immense strides taken in journalistic literature in the past forty years, both in quantity and in quality, from the one journal of forty years ago, to the twenty-one of to-day. He urged that every man should keep a record of every step in advance. Let those who are not satisfied with our present literary status try to make it better—no one would hinder. He regretted that there were no elementary books; books fit only for experienced professional men were all that we had for students. Any one who would prepare graded books for dental students would achieve a great desideratum. The nomenclature of anatomy and pathology should also be anglicized. Students require aids to rapid progress.

*Dr. J. J. R. Patrick*—Thought the student should be well grounded in elementary principles of science in the high schools before entering the colleges.

*Dr. J. R. Walker*—Thought elementary instruction in hygiene was needed in our primary schools,to enable students to pass through the high schools to the college with sound minds in sound bodies, success in after-life depending upon health of body as well as cultivation of brain.

*Dr. E. S. Chisholm*—Our literature is what we make it. The supply is evolved from the demand.

*Prof. J. Taft*—Elementary science, as taught in high schools, gives a mere smattering. Graded text books are needed. Prof. Ford, and others, have prepared question books as guides to study, taking the present text-books as statements of facts to be discovered by the aid of the question books.

*Dr. Spalding*—Much that is now contained in the text-books is erroneous from a modern standpoint. We must draw from our own ranks, and train our own authors.

*Dr. Patrick*—The present standard works are designed for the general practitioner. They are not guides to any specialty, as of the eye, the ear, or the teeth.

*Dr. A. W. Harlan*—Dental students are not school-boys to be taught mere rudiments. We have books enough, but we do not study them. There is too great a multiplication of primers already. What we need is modern revisions of our present works, and a closer study of them.

*Dr. B. H. Teague*—Moved the recognition by the Association of the work of "Mrs. M. W. J.," entitled "Letters from a Mother to a Mother," a work of national reputation and world-wide fame.

*Prof. J. Taft*—Moved, in addition, the recommendation of the book to all practicing dentists, for general circulation.

*Dr. C. W. Spalding*—The work is the best of its class. It has no equal for placing in the hands of mothers.

The motion as amended was passed unanimously.

"Mrs. W. M. J." was introduced to the Association, and tendered her thanks for the compliment paid.

The subject of Dental Literature was passed.

Dr. McKellops moved the discussion of the subject of Dental Ethics, condemning the signing of certificates to the merits of secret preparations, as an improper mode of advertising a professional man's name and address.

Nashville, Tenn., was selected as the next place of meeting, and the following named members were elected to serve as officers for the ensuing year:

*President*—Dr. W. C. Wardlaw, Augusta, Ga.

*First Vice-President*—Dr. B. H. Catching, Atlanta, Ga.

*Second Vice-President*—Dr. J. Rollo Knapp, New Orleans, La.

*Third Vice-President*—Dr. E. D. Hamner, Galveston, Texas.

*Cor. Secretary*—Dr. E. S. Chisholm, Tuscaloosa, Ala.

*Rec. Secretary*—Dr. R. A. Holliday, Atlanta, Ga.

*Treasurer*—Dr. H. A. Lowrance, Athens, Ga.

*Executive Committee*—Dr. G. F. S. Wright, Columbia, S. C.; Dr. W. H. Morgan, Nashville, Tenn.; Dr. W. H. Richards, Knoxville, Tenn.

"Mrs. M. W. J." was elected an honorary member of the Association.

The thanks of the Southern Dental Association were tendered to the Faculty of the Tulane University and the Board of Management; to the S. S. White Co., through Mr. J. W. Selby; to the daily press of New Orleans; to the retiring President, Dr. A. O. Rawls, and to the Secretary and Treasurer; to the dentists of New Orleans, and to the Board of Management of the World's Exposition.

The Association then adjourned to meet on the last Tuesday in May, 1886, at Nashville, Tenn.

